# Supplementary material for: The Evolution and Characterization of the RNA Interference Pathways in Lophotrochozoa
Source: Genome Biol Evol. 2024 May 7;16(5):evae098. doi: 10.1093/gbe/evae098 (PMC11114477; doi:10.1093/gbe/evae098)
Supplement: evae098_Supplementary_Data [file evae098_supplementary_data.zip › Supplementary_materials.zip!/selected_miRNAs/NoGe_selected_miRNAs/mirdeep_pdf_files/NMRB01000004.1_Notospermus_geniculatus_strain_Ushimado_scaffold4,_whole_genome_shotgun_sequence_162.pdf]

Provisional ID	:	NMRB01000004.1_Notospermus_geniculatus_strain_Ushimado_scaffold4,_whole_genome_shotgun_sequence_162
Score total	:	482.6
Score for star read(s)	:	3.9
Score for read counts	:	477.3
Score for mfe	:	2
Score for randfold	:	0
Score for cons. seed	:	-0.6
Total read count	:	948
Mature read count	:	886
Loop read count	:	0
Star read count	:	62

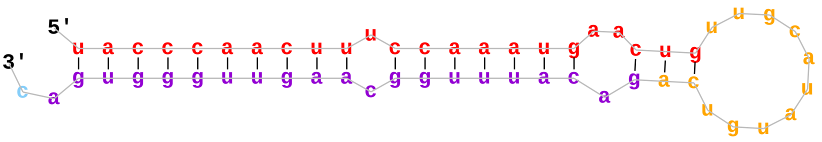

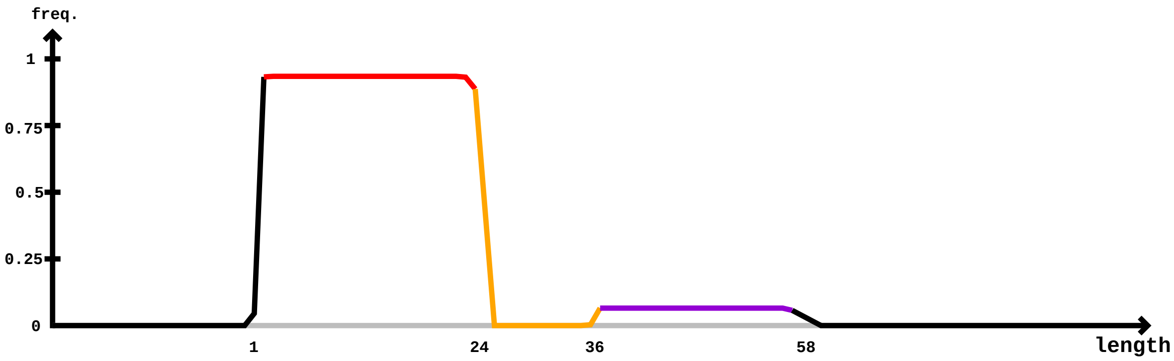


```
                          Mature                            Star
5'- ucacauuugccuagagcaguuacccaacuuuccaaaugaacuguugcauaugucagacauuuggcaaguugggugacuguuccaggauugaaaugucugacaguauaggggc   -3'   obs
    ucacauuugccuagagcaguuacccaacuuuccaaaugaacuguugcauaugucagacauuuggcaaguugggugacuguuccaggauugaaaugucugacaguauaggggc         exp
    ..((((((.(((.(((((((((((((((((.(((((((..(((..........))).))))))).))))))))))))))))).)))....))))))................   reads   mm   sample
    ...................uuacccaacuuuccaaaCgaacug.....................................................................   1       1    Fm3
    ...................uuacccaacuuuccaaaAgaacug.....................................................................   1       1    Fm3
    ...................uuacccaacuuuccaaaugaacug.....................................................................   9       0    Fm3
    ....................uacccaacuuuccaaaugaac.......................................................................   1       0    Fm3
    ....................uacccaacuuuccaaaugaacu......................................................................   8       0    Fm3
    ....................uaAccaacuuuccaaaugaacug.....................................................................   1       1    Fm3
    ....................uacccaacCuuccaaaugaacug.....................................................................   1       1    Fm3
    ....................uacccaacuuuccaaaGgaacug.....................................................................   1       1    Fm3
    ....................uacccaacuuuccaaauCaacug.....................................................................   1       1    Fm3
    ....................uUcccaacuuuccaaaugaacug.....................................................................   1       1    Fm3
    ....................uacccaacuuuccaaaugaacug.....................................................................   231     0    Fm3
    ....................uacccGacuuuccaaaugaacug.....................................................................   1       1    Fm3
    ....................uacccaacuuuccaaaugaacugu....................................................................   4       0    Fm3
    .....................acccaacuuuccaaaugaacug.....................................................................   1       0    Fm3
    ......................................................agacauuuggcaaguuggguga....................................   2       0    Fm3
    .......................................................gacauuuggcaaguuggguga....................................   3       0    Fm3
    .......................................................gacauuuggcaaguugggugU....................................   1       1    Fm3
    .......................................................gacauuuggcaaguugggugac...................................   10      0    Fm3
    .......................................................gacauuuggcaaguugggugaA...................................   1       1    Fm3
    .......................................................gacauuuggcaaguugggugacu..................................   5       0    Fm3
    .......................................................gacauuuggcaaguugggugacA..................................   2       1    Fm3

    ...................uuacccaacuuuccaaaugaacu......................................................................   1       0    Mm3
    ...................uuacccaacuuuccaaaugaacug.....................................................................   8       0    Mm3
    ....................uacccaacuuuccaaaugaacu......................................................................   4       0    Mm3
    ....................uacccaacuuuccaaaugaacug.....................................................................   29      0    Mm3
    ....................Cacccaacuuuccaaaugaacug.....................................................................   1       1    Mm3
    ....................uacccaacuuAccaaaugaacug.....................................................................   1       1    Mm3
    ....................uacccaacuuuccaaaugaacugu....................................................................   1       0    Mm3
    .......................................................gacauuuggcaaguugggug.....................................   1       0    Mm3
    .......................................................gacauuuggcaaguugggugac...................................   1       0    Mm3
    .......................................................gacauuuggcaGguugggugac...................................   1       1    Mm3
    .......................................................gacauuuggcaaguugggugacG..................................   1       1    Mm3
```

Mature Star

```
ucacauuugccuagagcaguuacccaacuuuccaaaugaacuguugcauaugucagacauuuggcaaguuggguga cuguuccaggauugaaaugucugacaguauaggggc
.......................................................gacauuuggcaaguugggugacu..................................   3    0   Mm3

....................uacccaacuuuccaaaugaacug.....................................................................   22   0   Fm1
.......................................................gacauuuggcaaguuggguga....................................   4    0   Fm1

...................uuacccaacuuuccaaaugaacug.....................................................................   15   0   Fm2
....................uacccaacuuuccaaaugaacug.....................................................................   29   0   Fm2
....................uacccaacuuuccaaauCaacug.....................................................................   1    1   Fm2
....................uacccaacuuuccaaauAaacug.....................................................................   1    1   Fm2
....................uacccaacuuuccaaaugaacuU.....................................................................   1    1   Fm2
....................uGcccaacuuuccaaaugaacug.....................................................................   1    1   Fm2
....................uacccaacuuuccaaaGgaacug.....................................................................   1    1   Fm2
.......................................................gacauuuggcaaguugggug.....................................   7    0   Fm2

....................uacccaacuuuccaaaugaacu......................................................................   6    0   Mm1
....................uacccaacuuucUaaaugaacug.....................................................................   1    1   Mm1
....................uacccaacuuuccaaaCgaacug.....................................................................   1    1   Mm1
....................uacAcaacuuuccaaaugaacug.....................................................................   1    1   Mm1
....................uacccaacuuuccaaaugaacug.....................................................................   88   0   Mm1
.......................................................gacauuuggcaaguugAguga....................................   1    1   Mm1
.......................................................gacauuuggcaaguuggguga....................................   10   0   Mm1

...................uuacccaacuuuccaaaugaacu......................................................................   2    0   Mm2
...................uuacccaacuuuccaaUugaacug.....................................................................   1    1   Mm2
...................uuacccaacuuuccaaauCaacug.....................................................................   1    1   Mm2
...................uuacccaacuuuccaaaugaacug.....................................................................   4    0   Mm2
....................uacccaacuuuccaaauCaac.......................................................................   1    1   Mm2
....................uacccaacuuuccaaaugaac.......................................................................   1    0   Mm2
....................uacccaacuuuccaaaugaacu......................................................................   14   0   Mm2
....................uacccaacuuuccaaauCaacu......................................................................   7    1   Mm2
....................uacccaacuuuccaaaugUacug.....................................................................   1    1   Mm2
....................uacccaacuuGccaaaugaacug.....................................................................   1    1   Mm2
....................uacccaacuuuccaaauAaacug.....................................................................   2    1   Mm2
....................uacccaacuuuccaaauCaacug.....................................................................   3    1   Mm2
....................uacccaacuuuccaaauUaacug.....................................................................   2    1   Mm2
....................uacccaacuuuccaGaugaacug.....................................................................   1    1   Mm2
....................uacccaacuuuUcaaaugaacug.....................................................................   1    1   Mm2
....................uGcccaacuuuccaaaugaacug.....................................................................   3    1   Mm2
....................uacccaacuuuccaaaAgaacug.....................................................................   1    1   Mm2
....................uacccaacuuuccaaaugaacug.....................................................................   352  0   Mm2
....................uUcccaacuuuccaaaugaacug.....................................................................   1    1   Mm2
....................uacccaacuuuccaaaCgaacug.....................................................................   4    1   Mm2
....................uacccaacGuuccaaaugaacug.....................................................................   1    1   Mm2
....................uaccAaacuuuccaaaugaacug.....................................................................   2    1   Mm2
....................uacccGacuuuccaaaugaacug.....................................................................   1    1   Mm2
....................uacccaacuuucUaaaugaacug.....................................................................   1    1   Mm2
....................uacccaacuuuccaaaugaacugu....................................................................   2    0   Mm2
.....................acccaacuuuccaaaugaacug.....................................................................   1    0   Mm2
......................................................agacauuuggcaaguugggugU....................................   1    1   Mm2
.......................................................gacauuuggcaaguuggguga....................................   3    0   Mm2
.......................................................gacauuuggcaaguugggugac...................................   1    0   Mm2
.......................................................gacauuuggcaaguugggugacA..................................   1    1   Mm2
.......................................................gacauuuggcaaguugggugacu..................................   3    0   Mm2
```
